# A Rainfall- and Temperature-Driven Abundance Model for *Aedes albopictus* Populations

**DOI:** 10.3390/ijerph10051698

**Published:** 2013-04-26

**Authors:** Annelise Tran, Grégory L’Ambert, Guillaume Lacour, Romain Benoît, Marie Demarchi, Myriam Cros, Priscilla Cailly, Mélaine Aubry-Kientz, Thomas Balenghien, Pauline Ezanno

**Affiliations:** 1 CIRAD, UPR AGIRs, Montpellier F-34398, France; E-Mail: p.cailly@laposte.net; 2 CIRAD, UMR TETIS, Montpellier F-34398, France; 3 EID Méditerranée, Montpellier F-34184, France; E-Mails: glambert@eid-med.org (G.L.A.); glacour@eid-med.org (G.L.); rbenoit@eid-med.org (R.B.); mcros@eid-med.org (M.C.); 4 Biodiversity Research Center, Earth and Life Institute, Université Catholique de Louvain, Louvain-la-Neuve B-1348, Belgium; 5 SIRS, Montpellier F-34093, France; E-Mail: marie.demarchi@sirs-fr.com; 6 INRA, UMR1300 Biologie, épidémiologie et analyse de risques en santé animale, Nantes F-44307, France; E-Mails: melaine.aubry.kientz@gmail.com (M.A.-K.); pauline.ezanno@oniris-nantes.fr (P.E.); 7 ONIRIS, LUNAM Université Nantes Angers Le Mans, Nantes F-44307, France; 8 CIRAD, UMR CMAEE, Montpellier F-34398, France; E-Mail: thomas.balenghien@cirad.fr

**Keywords:** *Aedes albopictus*, arbovirus, population dynamics, modelling, sensitivity analysis

## Abstract

The mosquito *Aedes (Stegomyia) albopictus* (*Skuse*) (*Diptera: Culicidae*) is an invasive species which has colonized Southern Europe in the last two decades. As it is a competent vector for several arboviruses, its spread is of increasing public health concern, and there is a need for appropriate monitoring tools. In this paper, we have developed a modelling approach to predict mosquito abundance over time, and identify the main determinants of mosquito population dynamics. The model is temperature- and rainfall-driven, takes into account egg diapause during unfavourable periods, and was used to model the population dynamics of *Ae. albopictus* in the French Riviera since 2008. Entomological collections of egg stage from six locations in Nice conurbation were used for model validation. We performed a sensitivity analysis to identify the key parameters of the mosquito population dynamics. Results showed that the model correctly predicted entomological field data (Pearson r correlation coefficient values range from 0.73 to 0.93). The model’s main control points were related to adult’s mortality rates, the carrying capacity in pupae of the environment, and the beginning of the unfavourable period. The proposed model can be efficiently used as a tool to predict *Ae. albopictus* population dynamics, and to assess the efficiency of different control strategies.

## 1. Introduction

The Asian tiger mosquito *Aedes (Stegomyia) albopictus* (*Skuse*) (*Diptera: Culicidae*) is a competent vector for several arboviruses, such as dengue and chikungunya viruses [1]. Originally indigenous to South-East Asia, the species has spread during the last decades to Africa, the Middle East, Europe and the Americas [2]. Since 1990, the Asian tiger mosquito has rapidly colonized Southern Europe [3]. It is the only invasive mosquito species currently installed in continental France, present since 2004 on the Côte d’Azur region [4,5,6]. Its continuing spread is of increasing public health concern, strengthened by a recent chikungunya outbreak in Italy in 2007 [7], and the first occurrence of autochthonous dengue and chikungunya cases in southern France in 2010 [8,9]. 

Due to the urgent need for intensive monitoring and risk-based surveillance of *Ae. albopictus* populations, modelling approaches have been used to map the areas of its potential distribution [10,11]. Moreover, mechanistic modelling approaches have been successfully used to predict the temporal dynamics of *Ae. aegypti* using either stochastic [12] or deterministic models [13], or discuss the impact of mosquito population dynamics on chikungunya virus transmission to the human population on La Réunion island, France [14,15]. Such models are useful to identify the control points of the population dynamics, and test some control strategies [14]. In Europe, Poletti *et al.* recently modelled the temporal dynamics of *Ae. albopictus* in Italy and discussed the transmission potential of chikungunya virus [16]. 

In this paper, we present a weather-driven abundance model depicting the annual and inter-annual variations of *Ae. albopictus* populations taking into account diapause processes. We used the generic framework proposed recently by Cailly *et al.* [17] for modelling mosquito populations, and we defined parameters and functions to adapt this generic model to the Asian tiger mosquito. The model was used to simulate *Ae. albopictus* populations in the Côte d’Azur region, using daily rainfall and temperature data. Entomological collections of egg stage from different municipalities were used for model validation, and a sensitivity analysis was performed to identify the key parameters driving the mosquito population dynamics.

## 2. Material and Methods

### 2.1. Study Area

**Figure 1:**
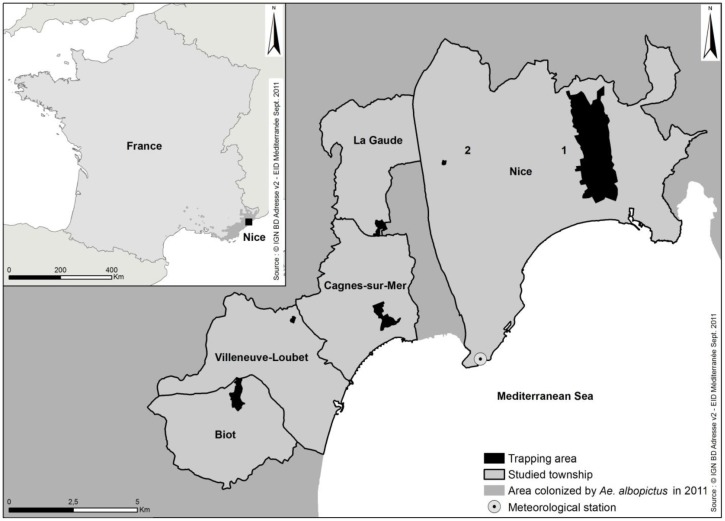
Study area including the municipalities of Nice, La Gaude, Cagnes-sur-Mer, Villeneuve-Loubet, and Biot. Source: © IGN BD Adresse v2—EID Méditerranée, September 2011.

The study area includes the municipality of Nice and neighbouring municipalities from the French Riviera region where entomological longitudinal surveys on *Ae. albopictus* populations are performed since 2008 (Figure 1). In this region located in South Eastern France, the climate is typically Mediterranean, with warm, very dry summers and mild, wet winters. Total annual rainfall is around 750 mm, and temperatures usually vary between 0 °C in winter, and 34 °C in summer. *Aedes albopictus* populations are mainly installed in urban areas, as in Nice, a densely populated city with a population over 340,000 inhabitants, and in the residential areas of neighboring municipalities. 

### 2.2. Entomological Data

Mosquito sampling was performed in different locations of Nice conurbation, using ovitraps’ networks placed mostly in sites shaded by vegetation. The surveillance of eggs was chosen for detecting invasive mosquito species such as *Ae. albopictus* in South-eastern France. It allows targeted and rapid sampling efforts and an optimal cost-benefit ratio [5,18]. Ovitraps are artificial egg-laying containers made up with 3 L black plastic buckets (Dillewijn Zwapak^©^, Aalsmeer, The Netherlands) filled with 2 L of tap water and the biolarvicide *Bacillus thuringiensis israelensis* (Bti) to prevent the production of mosquitoes in the trap [19], in which a floating polystyrene square (5 × 5 cm) was added to provide a support for oviposition. The sampling was conducted in six different locations of Nice conurbation, mainly in discontinuous urban fabric including individual houses with gardens: Nice (1: Nice downtown, 2: residential area on the outskirts of Nice), Cagnes-sur-Mer, La Gaude, Biot, and Villeneuve-Loubet. Networks of between 15 and 50 ovitraps were collected biweekly, and weekly in Cagnes-sur-Mer (Table 1). We considered the fortnightly frequency as an acceptable compromise between representative data (average period of time separating two generations during summer, considering around three days for embryogenesis, seven days of larval development and five days for mating, blood engorgement and oviposition) and reasonable trapping effort in the five areas [20,21]. Surveys were stopped at the end of each year after two consecutive negative samples. Trapping areas were considered free of insecticide treatments realized during the plan of anti-dissemination of chikungunya virus from 2008 to 2011, except three ovitraps located in Nice-1 within a distance of 200 m from the insecticide spraying. Thus, results from these three ovitraps after the treatments were removed from the analysis. Hatched and unhatched eggs of *Ae. albopictus* were counted in laboratory under stereomicroscope. Eggs of *Ae.* (*Finlaya*) *geniculatus*, the only other mosquito species to lay eggs into ovitraps in south-eastern France, were morphologically discriminated from *Ae. albopictus* eggs using a stereomicroscope [22,23]. 

**Table 1:** Entomological collections for the surveillance of *Aedes albopictus*, Côte d’Azur area, France, 2008–2011.

Campaign		Trapping season		Ovitrap network			Result
Location	Year	Beginning	End	Nb traps	Surface of the trapping area (ha) *	Sampling frequency	Annual max. of the mean number of collected eggs per ovitrap per capture session
Nice 1	2008	25 Mar	8 Dec	30	328.7	biweekly	170
Nice 1	2009	16 Apr	9 Dec	50	517.7	biweekly	233
Nice 1	2010	15 Apr	2 Dec	50	517.7	biweekly	462
Nice 1	2011	21 Apr	14 Dec	50	517.7	biweekly	311
Cagnes-sur-Mer	2010	21 Jun	15 Nov	15	30.5	weekly	177
Cagnes-sur-Mer	2011	28 Mar	28 Nov	18	41.6	weekly	169
La Gaude	2010	16 Jul	8 Oct	22	17.9	biweekly	566
Biot	2010	16 Jul	8 Oct	25	40.3	biweekly	830
Villeneuve-Loubet	2011	6 May	30 Nov	15	3.8	biweekly	1,108
Nice 2	2011	11 May	18 Nov	15	2.7	biweekly	654

* computed as the surface of the smallest polygon including all ovitraps with a buffer distance of 50 m.

### 2.3. Environmental Data

Daily rainfall and temperature data from 2008 to 2011 recorded in Nice Airport were obtained from the national meteorological service “Météo France”. Indeed, we assumed that the population dynamics of *Ae. albopictus* is mainly driven by these two factors: (*i*) temperatures have a strong impact on the survival of *Ae. albopictus* populations, and on the development of aquatic stages [20,24,25]; (*ii*) precipitations favor the availability of breeding sites, *i.e.*, any small recipient filled with water where *Ae. albopictus* females lay their eggs. Moreover, we considered that the egg hatching is triggered by rainfall events but also by human water supply (e.g., garden watering in summer) [20]. Indeed, larval surveys carried out since 2008 showed the importance of small and medium containers sampled in gardens in the productivity *Ae. albopictus*’ populations [26].

### 2.4. Model Description

#### 2.4.1. *Aedes albopictus* Life Cycle

As for all mosquito species, the life cycle of *Ae. albopictus* includes three water-dependent stages (egg, larva, and pupa), and one aerial stage (adult). The lifespan in each stage depends on several factors, such as temperature, or water availability. After emergence and insemination, female adults successively: (*i*) seek a human host to take a blood meal; (*ii*) rest in a sheltered place during the few days needed for the eggs to mature; and (*iii*) search for sites to lay their eggs. The Asian tiger mosquito breeds in artificial containers of any type (metal, glass, stone, plastic, rubber, *etc.*), or in small natural water bodies such as tree holes or rock pools [27]. Eggs hatch after a desiccation period (few days to several months) when they are submerged in water by rainfall or artificial flooding. The larvae then mature through four stages before entering pupation. Adult mosquito emerges from the pupa at the surface of water. In temperate climates, *Ae. albopictus* survive the unfavorable period (winter) as eggs in dormancy (diapause) that will hatch during the next favorable season (spring).

#### 2.4.2. Modelling *Aedes albopictus* Population Dynamics

**Figure 2:**
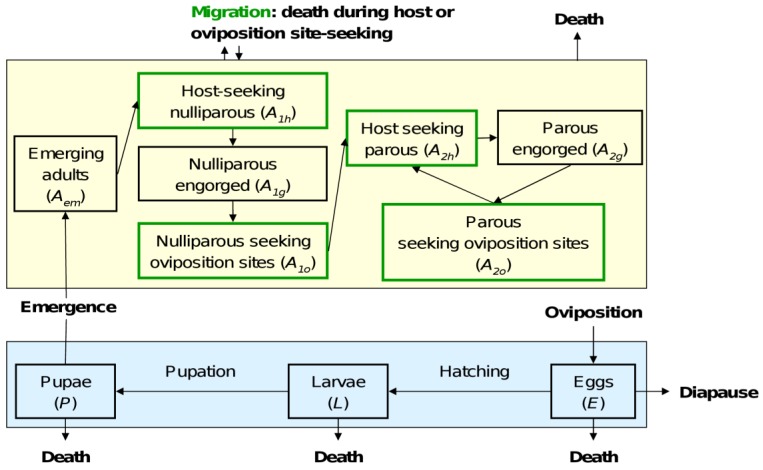
Model diagram of *Aedes albopictus* population dynamics in temperate climate. Aquatic stages are drawn in blue, adult females in yellow. The green compartments indicate the adult females which move to seek for a host or an oviposition site. Adapted from Cailly *et al.* [17]. Reprinted from Ecological Modelling, 227/24 February 2012, Cailly, P.; Tran, A.; Balenghien, T.; L’Ambert, G.; Toty, C.; Ezanno, P., A climate-driven abundance model to assess mosquito control strategies, Pages 7–17, Copyright (2012), with permission from Elsevier.

The generic model of mosquito population dynamics developed by Cailly *et al.* [17] represents all of the steps of the mosquito life cycle (Figure 2). It considers ten different stages: three aquatic stages (*E*, eggs; *L*, larvae; *P*, pupae), one emerging adult stage (*A_em_*), three nulliparous stages (*A_1h_*, *A_1g_*, *A_1o_*), and three parous stages (*A_2h_*, *A_2g_*, *A_2o_*). In the adult stage, females only are represented. Parous females are females that have oviposited at least once, whereas nulliparous females have never laid eggs. Adults are subdivided regarding their behaviour during the gonotrophic cycle (h, host-seeking; g, transition from engorged to gravid; o, oviposition site seeking). Once parous, females repeat their gonotrophic cycle until death. The events driving the transitions between stages are: egg mortality or hatching, larva mortality, pupation (moult of larvae to pupae), pupa mortality, adult emergence, mortality, engorgement, egg maturing, and oviposition. The model takes into account density-dependent mortality of the larval stage [28], and pupa density-dependent success of adult emergence. Density-dependent mortality was assumed at the larval stage as it is has been often observed [28,29]. Pupa density-dependent success of adult emergence was assumed as emergence success was found negatively correlated to pupa density [30].

The model is based on a system of ordinary differential equations (ODE). For *Aedes* populations in temperate climate, the eggs stop hatching at the beginning of the unfavorable period, during which diapause occurs. All other stages will continue their development or transition to the next stage. Thus, the ODE system is:

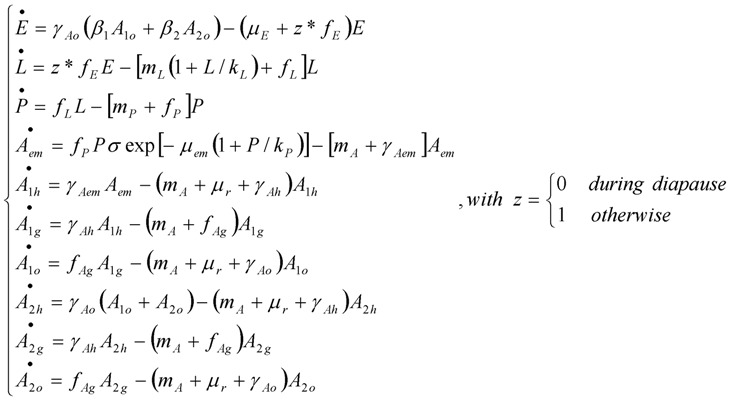
(1)

Model parameters are in Greek letters. They are constant. For stage X, *γ_X_* is the transition rate to the next stage, *μ_X_* the mortality rate, and *β_X_* the egg laying rate. *σ* is the sex-ratio at the emergence. *μ*_r_ is an additional adult mortality rate related to the seeking behavior, applied only on adult stages involving risky movements (host or oviposition site seeking). 

Model functions are in Latin letters. They depend on parameters and weather-driven functions (*i.e.*, functions of temperature, humidity or precipitation varying over time). For stage X, *f_X_* is the transition function to the next stage, *m_X_* the mortality function, and *k_X_* the environment’s carrying capacity which limits the population growth due to density-dependent processes. 

#### 2.4.3. Parameters and Functions of the Model

We defined specific parameter values, forcing functions, transition functions between stages of the life cycle, and mortality functions to adapt the model to *Ae. albopictus* in the region of Nice. As our model neglects mosquito dispersal (arrival or departure of individuals), the dimensions of the surface used for simulation have to be larger than the active flight distance of mosquitoes: *Ae. albopictus* having a distance of dispersal of about 100 m [31], our model is used for simulating the mosquito population dynamics over a squared surface greater than 4 ha.

Because of the large phenotypic variability of the Asian tiger mosquito [27], parameter values were based on expert knowledge of local *Ae. albopictus* population biology [25,26,32], supported and completed by scientific literature (Table 2).

**Table 2:** Parameter values of the model of mosquito population dynamics adapted to *Aedes albopictus* in Mediterranean temperate climate.

Parameter	Definition	Value	Reference
β_1_	Number of eggs laid by ovipositing nulliparous females (per female)	95	[25]
β_2_	Number of eggs laid by ovipositing parous females (per female)	75	[25]
κ_L_	Standard environment carrying capacity for larvae (larvae ha^−1^)	250,000	To our best knowledge
κ_P_	Standard environment carrying capacity for pupae (pupae ha^−1^)	250,000	To our best knowledge
σ	Sex-ratio at the emergence	0.5	[24]
μ_E_	Egg mortality rate (day^−1^)	0.05	(Lacour, unpublished)
μ_L_	Minimum larva mortality rate (day^−1^)	0.08	(Lacour, unpublished)
μ_P_	Minimum pupa mortality rate (day^−1^)	0.03	(Lacour, unpublished)
μ_em_	Mortality rate during adult emergence (day^−1^)	0.1	(Lacour, unpublished)
μ_A_	Minimum adult mortality rate (day^−1^)	0.02	[25]
μ_r_	Adult mortality rate related to seeking behavior (day^−1^)	0.08	To our best knowledge
T_E_	Minimal temperature needed for egg development (°C)	10.4	[24]
TDD_E_	Total number of degree-day necessary for egg development (°C)	110	(Lacour, unpublished)
γ_Aem_	Development rate of emerging adults (day^−1^)	0.4	To our best knowledge
γ_Ah_	Transition rate from host-seeking to engorged adults (day^−1^)	0.2	To our best knowledge
γ_Ao_	Transition rate from oviposition site-seeking to host-seeking adults (day^−1^)	0.2	To our best knowledge
T_Ag_	Minimal temperature needed for egg maturation (°C)	10	[24]
TDD_Ag_	Total number of degree-days necessary for egg maturation (°C)	77	[24]
t_start_	Start of the favorable season	10 Mar	[32]
t_end_	End of the favorable season	30 Sept	[32]

The end of the favorable period is defined as the moment when 90% of eggs laid enter into diapause. Diapause is genetically programmed in *Ae. albopictus* populations. It is induced by a short photoperiod [27]. In Nice area, egg diapause initiation occurs gradually during September, so diapause processes occur in the model from 30 September to 10 March the following year [32]. During this period, only eggs survive until the start of the next favorable season when they hatch if they are immerged in water. The standard environment carrying capacities for larvae (*κ_L_*) and pupae (*κ_P_*) were estimated as follows: the maximal larval and pupal densities observed in laboratory (10 individuals per cm² of water surface [33]) was multiplied by the surface of a typical *Ae. albopictus* breeding site (~50 cm²), the number of breeding sites per household (~20), and the number of households per hectare (~25), all of these values being estimated from field observations [26].

The two forcing function variables are temperature (T) and precipitation (P), both varying over time. Daily mean temperature and precipitation were used. Precipitation is known to trigger egg hatching of *Aedes* species breeding in out-door oviposition sites [34]. However, in human-made environments artificial flooding (e.g., watering of gardens) is likely to be the main driver of *Ae. albopictus* egg hatching in summer, when rainfall events are scarce. Thus, we considered that the development of *Ae. albopictus* eggs is driven by: (*i*) the occurrence of rainfall events in spring; and (*ii*) temperature, using the concept of degree-day, the quantity of accumulated heat necessary for development from one stage to another. In spring, hatching occurs only with rainfall events: the transition function from egg to larva *f_E_*(*t*) will be null if no rainfall event occurs at time *t* (*P*(*t*) = 0). Otherwise, it is driven by temperature, using the degree-day relation, also used to express the development rate of engorged adults becoming gravid at time *t*:

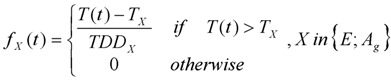
(2)

Values of T_X_ and TDD_X_ are given in Table 2. 

The development of other aquatic stages (larvae and pupae) is positively correlated to temperature within an optimum range [24]. Non-linear relations were used to express the relationships between temperature (in °C) and development rate of larvae and pupae according to observations under laboratory controlled conditions [24]. These functions are given in Table 3. It should be noted that atmospheric temperature was used as a proxy of water temperature. This approximation is justified by the small size of the urban breeding sites of *Ae. albopictus* in Nice region*.*

**Table 3:** Functions of the model of mosquito population dynamics adapted to *Aedes albopictus* in Mediterranean temperate climate.

Function	Definition	Expression
*f_E_*	Transition function from egg to larva	Equation (2)
*f_L_*	Transition function from larva to pupa	f_L_(t) = −0.0007. T²(t) + 0.0392. T(t) − 0.3911
*f_P_*	Transition function from pupa to emerging adult	f_P_(t) = 0.0008. T²(t) − 0.0051. T(t) + 0.0319
*f_Ag_*	Transition function from engorged adult to oviposition site—seeking adult	Equation (2)
*m_L_*	Larva mortality (day^−1^)	m_L_(t) = exp(−T(t)/2) + μ_L_
*m_P_*	Pupa mortality rate (day^−1^)	m_P_(t) = exp(−T(t)/2) + μ_P_
*m_A_*	Adult mortality rate (day^−1^)	m_A_(t) = max(μA; 0.04417 + 0.00217. T(t))
*k_L_*	Environment carrying capacity of larvae (ha^−1^)	Equation (3)
*k_P_*	Environment carrying capacity of pupae (ha^−1^)	Equation (3)

Temperature also impacts the mortality rates of larvae, pupae and adults. Expressions were derived from Shaman *et al.* [35], considering one different function for each stage, and adapted to the observations of *Ae. albopictus* [24] (Table 3).

Finally, we considered that the precipitations impact the environment’s carrying capacity (*k_X_*) of aquatic stages (larvae and pupae), increasing the number of breeding sites available for *Ae. albopictus* which is essentially an outdoor breeder [36,37,38]:

*k_X_ (t)= κ_X_* × (P_norm_(t)+1), *X* in {*L*;*P*} (3)

Values of *κ_X_*, the standard environment’s carrying capacity of aquatic stages, are given in Table 2. *P_norm_*(*t*) is defined as the rainfall amount summed over a two weeks period, and normalized in order to vary between 0 and 1.

#### 2.4.4. Model Outputs

The model predicts the abundance of mosquitoes per stage (*E*, *L*, *P*, *A_em_*, *A_1h_*, *A_1g_*, *A_1o_*, *A_2h_*, *A_2g_*, *A_2o_*) over time. In addition, the dynamic information computed by the model was aggregated using average output values following Cailly *et al.* [17]: the adult peak (maximum number of adults observed in a year), the attack rate (average of the daily number of host-seeking adults (*A_h_* = *A_1h_* + *A_2h_*) during the 21 days around the peak dates), and the parity rate (ratio of the total number of parous females to the total number of females). These aggregated outputs were chosen because of their epidemiological importance. They will be used to perform the sensitivity analysis of the model (Section 2.6). Finally, to allow the comparison between the entomological collections from the ovitraps (as described in Section 2.2) and the predictions of the model, the abundance of eggs laid at time *t* by *Ae. albopictus* females, *E_l_*(*t*), was computed as:

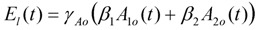
(4)

#### 2.4.5. Initial Conditions and Simulations

The differential equations were discretized using the explicit Euler method that we implemented in Scilab 5.1 [39]. Simulations were run over five years (2008–2011, and one first year with average values of precipitation and temperature, not retained for output computation). Initially, the population consisted of 10^6^ eggs (stage E), *t0* corresponding to 1 January.

### 2.5. Validation

Because the collected eggs in ovitraps are removed after sampling, we compared the observed average number of eggs per trap (relative to the maximum value of the observed average number of eggs per trap) in each site (Nice-1 and -2, Cagnes-sur-Mer, La Gaude, Biot, and Villeneuve-Loubet) with the simulated abundances of eggs newly laid (*E_l_*) (relative to the maximum value of simulated eggs abundance over the 2008–2011 period). The degree of association between observed and simulated number of eggs at the time of ovitrap collection was assessed for each collection site by calculating the Bravais–Pearson correlation coefficient.

### 2.6. Sensitivity Analysis

We carried out a global sensitivity analysis, varying simultaneously all of the model’s parameters described in Table 2 (20 parameters) using a fractional factorial design [40]. Such a design enabled us to estimate the sensitivity indices for the principal effects and the first-order interactions between parameters (3 levels per factor: the nominal value ±10%, generating 2,186 scenarios). Based on the model realizations implemented in this design, the contributions of the variation factors to the output variability were evaluated using a linear regression approach [40]. For each aggregated output (as described in Section 2.4.4), a linear regression model was fitted with all the principal effects of the factors and their first-order interactions. A minimum variance criterion was defined: factors or interactions accounting for more than 1% of the output variance were retained in the model. The contribution of factor or interaction *i* to the variation in output *y* was the ratio of the sum of squares related to *i* on the total sum of squares of the model for output *y*. The sum of the contributions for output *y* equaled the coefficient of determination of the regression model *r*².

## 3. Results and Discussion

### 3.1. Aedes albopictus Population Dynamics in Urban Areas of South Eastern France

The dynamics of *Ae. albopictus* populations in the Côte d’Azur area present a strong seasonal variability with a 6-month period of adult activity and a 6-month period of egg diapause. The first eggs of the year are laid at the beginning of May. Mosquito density is maximal early July, late August and early September, depending on years and cities. Oviposition activity decreases between mid-September and mid-October, with a residual egg laying activity remaining until November or December. Sampling results did not show evidence of continuous oviposition activity during winter, unlike the situation reported in Rome, Italy [41].

**Figure 3:**
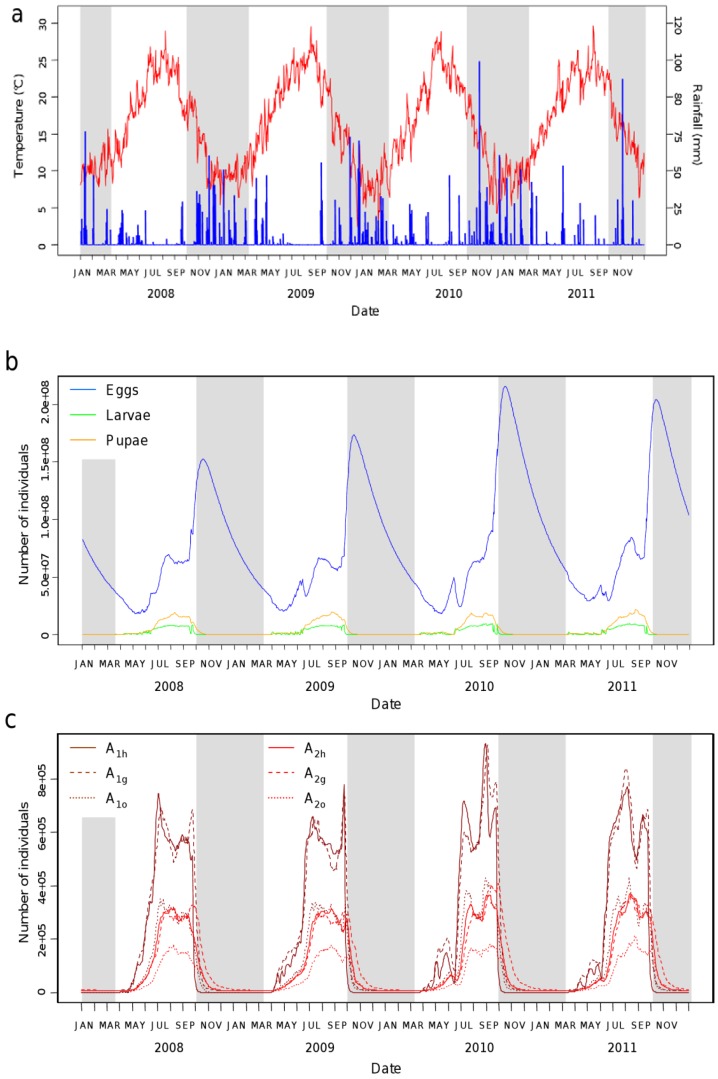
*Aedes albopictus* population dynamics simulated over four years according to temperatures and rainfall, Nice region, 2008–2011. (**a**) Daily mean temperature (red) and rainfall (blue). (**b**) Simulated number of individuals in the aquatic stages (eggs, larvae and pupae). (**c**) Simulated number of individuals in the aerial stages (*A_1h_*: nulliparous host-seeking; *A_1g_*: nulliparous engorged; *A_1o_*: nulliparous seeking oviposition site; *A_2h_*: parous host-seeking; *A_2g_*: parous engorged; *A_2o_*: parous seeking oviposition site). The alternation of favorable and unfavorable periods is represented in white and grey, respectively.

Based on observed temperatures and precipitations from 2008 to 2011, the model showed *Ae. albopictus* adult mosquitoes to be present in Nice from May to November with a maximum population in late August–early September (Figure 3). The number of eggs reaches a maximum at the beginning of the unfavorable period, when eggs stop hatching while adult females continue oviposition activity. The egg reserve decreases during winter time, allowing the survival of the population. Differences between years were due to differences in weather variables, the model being otherwise deterministic.

### 3.2. Model Validation

**Figure 4:**
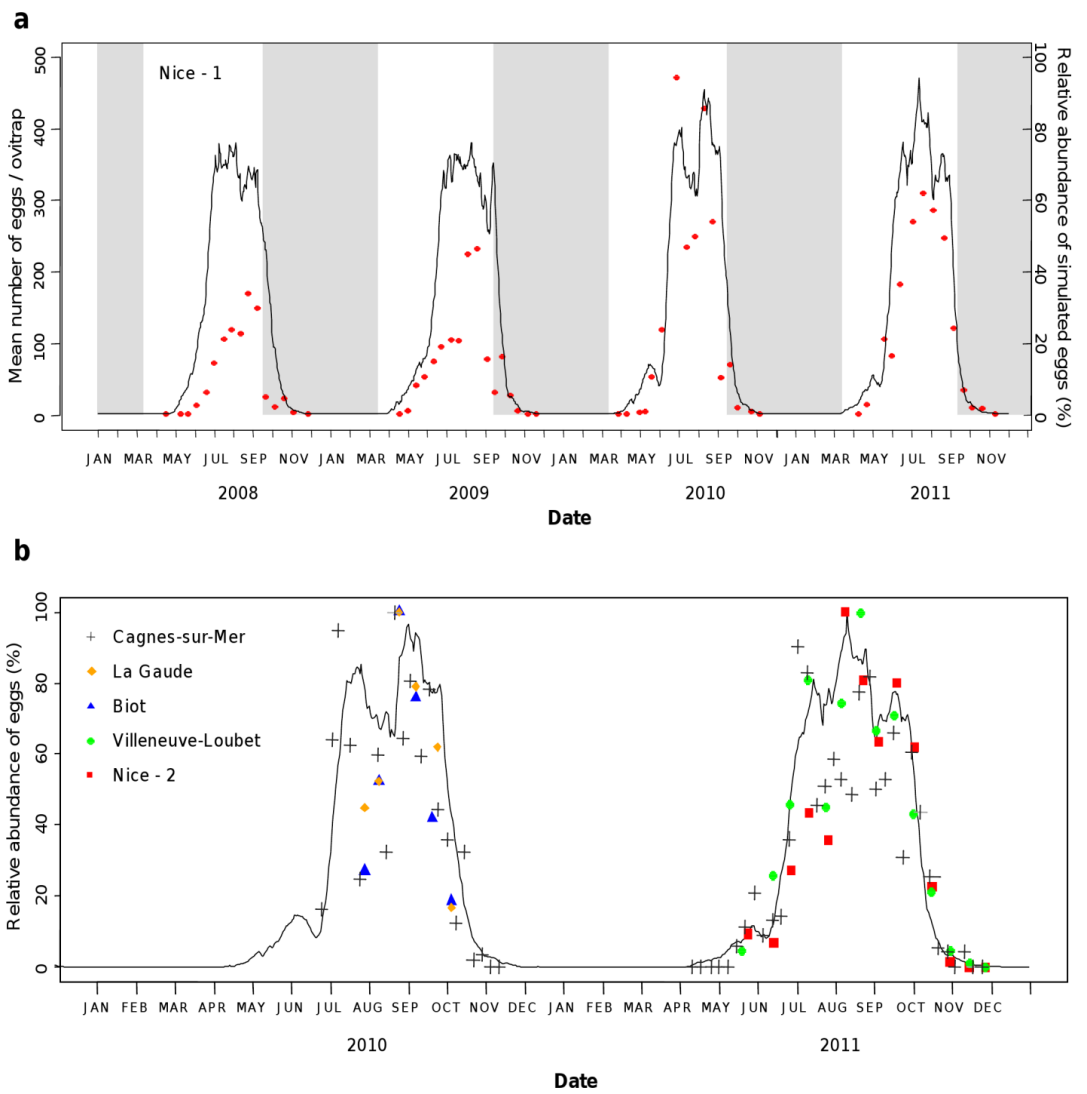
Model validation. (**a**) The simulated dynamics of eggs laid at time t *E_l_*(*t*) (black line) based on observed temperatures and precipitations from 2008 to 2011 was compared to the mean number of eggs collected per ovitrap in Nice-city area (red dots) using relative abundances. The alternation of favorable and unfavorable periods is represented in white and grey, respectively. (**b**) Simulated and observed eggs abundances, Nice area, 2010–2011. Symbols represent the observed mosquito abundance data in the different sites, and the black line is the simulated mosquito abundance.

Simulated mosquito abundances were highly consistent with field data collected in Nice-1, 2008–2011 (Figure 4(a)), with a cross-correlation value *r* = 0.79. For the four years under consideration the model reproduces well the abundance peak of catches occurring in late August. When considering 2010 and 2011, for both years the model simulates well the population growth in May and its decline in autumn. Yet, the model overestimates the abundances of eggs at the start of the favorable period (April–July) in 2008 and 2009, and all over the year in 2008. In 2010 and 2011, egg abundances were also slightly overestimated in April and May. The model also correctly simulated the abundance of *Ae. albopictus* in the five other sites (Figure 4(b)), with correlation coefficients of 0.73 for Biot (2010), 0.77 for Cagnes-sur-Mer (2010–2011), 0.87 for La Gaude (2010), 0.92 for Nice-2 (2011), and 0.93 for Villeneuve-Loubet (2011). Altogether, these results demonstrate that our model nicely predicts the dynamics of *Ae. albopictus* in various environments of the urban areas of Nice area and for different years.

### 3.3. Key Model Parameters

**Figure 5:**
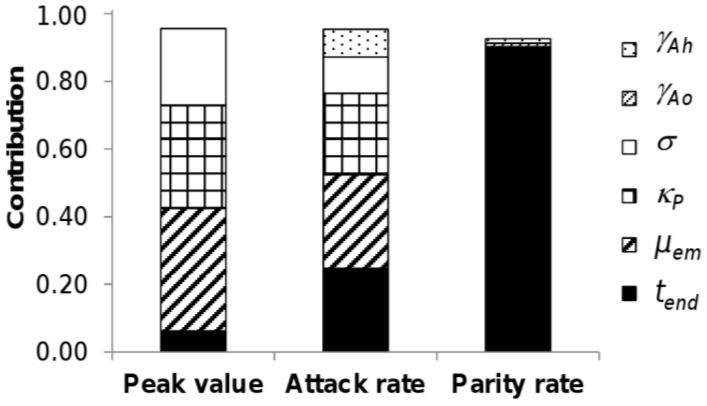
Contribution of model parameters to model output variance. Only parameters contributing to more than 1% of output variance were retained here. No interaction was retained.

The variations in the peak in adult abundance, in the attack rate and in the parity rate were mainly explained by six of the 20 parameters: the mortality rate at emergence (*μ_em_*), the carrying capacity in pupae of the environment (*κ_P_*), the end of the favourable period (*t_end_*), the sex-ratio (*σ*), the transition rate from host-seeking to engorged adults (*γ_Ah_*), and to a much lesser extent the transition rate from oviposition site-seeking to host-seeking adults (*γ_Ao_*) (Figure 5). 

**Figure 6:**
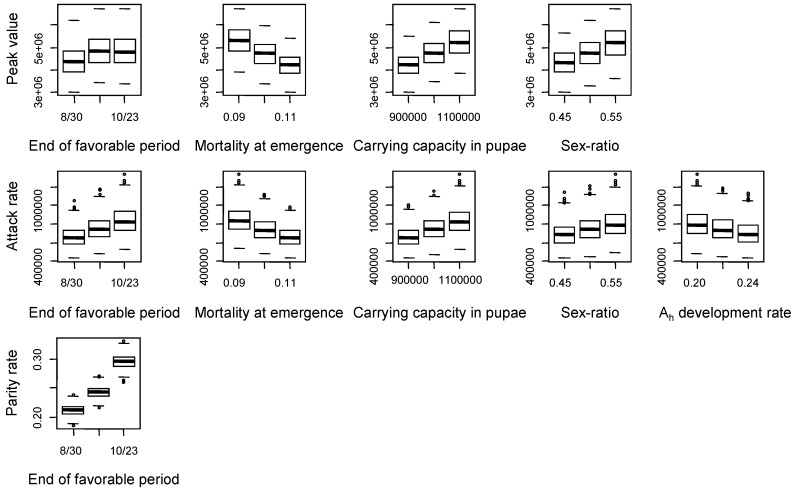
Variations of model outputs (in lines) with parameters contributing to more than 10% of their variance (in columns): 3 levels were tested per factor (nominal value ±10%). For each considered parameter and model output, a box-and-whisker diagram graphically depicts the maximum, minimum, median, lower and upper quartiles values of the model output obtained from the simulations with three different levels of the parameter tested.

Therefore, the better these parameters are known, the more precise the model will be in predicting these outputs. Further knowledge thus is needed concerning especially the mortality rate at emergence and the carrying capacity in pupae, which are quite uncertain parameters. A lower mortality at emergence, a higher carrying capacity in pupae, a longer favorable period, and a higher sex-ratio increased the peak abundance in adults and the attack rate (Figure 6). A shorter development of host-seeking adults decreased the attack rate. As expected, a longer favorable period also favored a higher parity rate.

### 3.4. Discussion

As far as we know, our model is the first mechanistic model of the dynamics of populations of *Ae. albopictus* in a temperate climate country taking into account diapause processes*.* Driven by two weather variables—temperature and rainfall—it describes the whole mosquito life cycle and takes into account egg diapause. Altogether, the simulations of the model were highly consistent with the number of eggs collected in ovitraps from six different sites of the Nice area over the 2008–2011 period. The model probably overestimates the populations of *Ae. albopictus* in 2008 and 2009 because at that time this invasive species was not yet fully established in Nice area [4].

These results clearly show that the underlying assumptions on the main drivers of Asian tiger mosquito dynamics in this region (*i.e.*, the impacts of temperature, rainfall, and artificial flooding) are correct, and that the model could be used to predict the dynamics of *Ae. albopictus* populations of following years. Yet, additional entomological data on adult populations and data originating from different cities of Southern France where the Asian tiger mosquito is now installed would strengthen confidence in the model predictions. The model could be applied on other Mediterranean areas where the Asian tiger mosquito is already installed and surveyed, such as Rome [42], Athens [43], Barcelona [44], and Tirana [45]. Moreover, it would be instructive to test the model in other temperate climates (United States of America or Japan) where European invasive populations of *Ae. albopictus* come from [46].

It should be noted that our model was easily adapted from a generic, mechanistic climate-driven model of mosquito populations developed by Cailly *et al.* [17]. Our results confirm the ability of Cailly’s model—applied on *Anopheles* species in [17]—to predict the dynamics of different species of mosquito populations, in different geographical areas and over several years. This model could be efficient and useful if used on other exotic mosquito invasive species established in European temperate areas: the Asian bush mosquito *Ae.* (*Finlaya*) *japonicus* (present since 2007 in northern Switzerland, 2008 in southern Germany and 2002 in Belgium), *Ae.* (*Finlaya*) *koreicus* (identified in 2008 in Belgium and 2011 in Italy), the yellow fever mosquito *Ae.* (*Stegomyia*) *aegypti* (established in Georgia, Abkhazia and Russia), and to a lesser extent *Ae.* (*Ochlerotatus*) *atropalpus* [3]. Indeed, these species have a similar biology with the Asian tiger mosquito: generally introduced by used tire trade, they breed in artificial containers and survive to cold winter temperature; they are human-biters in urban environments, increasing sanitary risk in regard as their proven or potential vector status [6].

Using a mechanistic approach, we can study the impact of temperatures and rainfall on the dynamics of *Ae. albopictus*, and our results are consistent with effects demonstrated in correlation studies [20]. Temperature is recognized to have a stronger influence on *Ae. albopictus* abundance than precipitation [47], and it is also the main driver of our model. Indeed, most of the mortality and transition rates are temperature-dependent functions. Temperature drives the mortality and transition rates functions in two different ways: higher temperatures favour higher transition rates between stages, although mortality rates decrease with temperature. Yet, according to our simulations in the Nice area, the impact of temperatures is rather favourable to *Ae. albopictus* populations: the peak of abundance occurs with the highest temperatures observed in summer (Figure 3).

**Figure 7:**
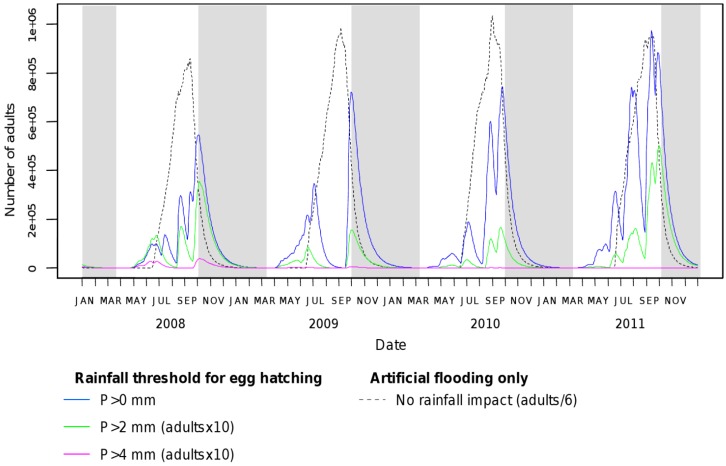
*Aedes albopictus* adult population dynamics with an egg hatching function driven either by rainfall or artificial flooding. Simulations were computed for different thresholds applied to daily rainfall for triggering egg hatching (P > 0 mm, P > 2 mm, and P > 4 mm).

On the other hand, previous observational studies stress the lack of clear relationship between precipitations and *Ae. albopictus* abundances, these studies showing either a positive effect of rainfall [38], a negative effect [20] or no effect at all [42]. Because *Ae. albopictus* females breed mainly in small artificial containers, Roiz *et al.* [20] suggest that the seasonal pattern of *Ae. albopictus* population dynamics might be more influenced by variations in human water supply than changes in precipitations. In this study, we followed this assumption to define egg hatching. In our model, this function is driven by rainfall in spring, and temperature-driven later on (Equation (2)), assuming that regular artificial flooding events trigger the egg hatching during the dry months of summer time. Our results stress the relevance and importance of this assumption in the urban environment of the Nice region. Indeed, simulations with an egg hatching function driven by rainfall the whole year (hatching occurring only with rainfall events) show that *Ae. albopictus* populations would be quasi absent during the summer months of dry years such as 2009, and that low rainfalls may lead to an extinction of the *Ae. albopictus* population (Figure 7). 

The sensitivity analysis identified six key parameters for the population dynamics model of *Ae. albopictus.* An improved knowledge of those parameters through laboratory or field studies will increase the precision of the model predictions. The sex-ratio of *Ae. albopictus* populations has been well studied [24]. The mortality rate at emergence measured in the field will have to be compared with laboratory data. The transition rates could be better estimated from laboratory studies under controlled temperature conditions. 

The sensitivity analysis demonstrates the importance for an invasive mosquito population in a temperate climate of the duration of the favorable period. To survive mosquito phenology must be adapted to their local environment, including a timely, appropriate initiation of the diapause process. *Aedes albopictus* diapause is a crucial adaptation, strengthening the survival capacity of the eggs to winter [48]. The diapause process is induced by a critical day length which is species- and location-dependent. This photoperiodism response is the main factor of adaptation of species to seasonal change. As compared to other studies on life history traits evolution in other animal species, the evolution in photoperiodism by *Ae. albopictus* is the fastest recorded [49]. Field studies on *Ae. albopictus* populations in Southern France showed that eggs enter diapause progressively between late August and mid-October [32]. The importance of the end of the favorable period, as emphasized by our results, suggests that the model could be modified to take into account this gradual entry in dormancy of *Ae. albopictus* populations. In the same way, the progressive hatching of diapausing eggs in spring [50] could be modeled. Furthermore, a different egg survival rate during both the favorable and unfavorable periods could be integrated in the model, to better simulate the incidences of diapause and weather harshness. Indeed, the modeled winter’s egg survivorship is lower than estimations in semi-controlled field studies [51,52]. 

The environment’s carrying capacity in larvae and pupae could be better estimated from field studies. These values depend on the number of available breeding sites in the field and on larval and pupal densities, which can reach up to 10 individuals per cm² in laboratory [33]. *Ae. albopictus* immature stages are mainly found in anthropogenic breeding sites, however there is an extreme spatial heterogeneity of breeding sites composition and abundance in the field. Field studies measuring different entomological indices (House, Breteau and Container indices [53,54]) suggest that the most suitable environment for the development of *Ae. albopictus* is a dense residential area combining individual houses and shaded gardens [55]. Therefore, information on the relationship between the type of urban land cover (residential areas with gardens, high density development areas, *etc.*) and the number of available breeding sites, complete with field measures of larva and pupa abundances, would help adapt the model according to land cover, with environment carrying capacities varying in time (with the rainfall) and in space. This would allow the development of a space-time population model, taking into account urban environment heterogeneity. 

The model’s parameters identified as the most influential could be the potential control points of the biological system. Hence, vector-control strategies achieving the modification of these parameters can be expected to influence notably the biting rate and therefore the associated risk of pathogen transmission to humans and animals. 

Chemical or biological treatments of larval instars, as well as the physical destruction of breeding containers will increase the mortality rate at emergence and decrease the environment’s carrying capacity in pupae. Obviously, adult mosquito control treatments will increase the mortality rate of adults. The use of repellents may raise the length of the host-seeking phase and, consequently, the related lethality.

Modifying the end date of the favorable period of activity for mosquito is *a priori* inconceivable. However, it is possible to mislead photosensitive stages by using night-interrupted light, thus avoiding diapause initiation and artificially decreasing winter survival of the populations. Previous experiments on mosquitoes and other insects like butterflies brought to light the photoinhibition of diapause as a way of control [56,57], but data on the efficiency and the applicability of such a method in a context of urban vector control is lacking.

To the best of our knowledge, there is no method to change the sex-ratio at the emergence. Yet, the impact of sterile insect technique (SIT), consisting in releasing sterile males which will compete with wild males for mating with females, could be modeled as a first approximation by diminishing the sex-ratio at the emergence. Indeed, females which have mated with a sterile male will have no offspring, and could be removed from the modeled adult population.

The primary application of our model is its use to elaborate and test effective control strategies against the Asian tiger mosquito. Indeed, there is currently no clearly-defined and efficient vector control strategy. All existing tools present problems for routine large scale applications [6]. Breeding sites can be physically removed to prevent the proliferation of *Ae. albopictus* populations. Yet, to be efficient such actions require repeated interventions and thus (*i*) the involvement of an important part of the vector control agencies’ workforce and (*ii*) a strong mobilisation of the population through an appropriate communication plan. SIT remains very expensive and better adapted to isolated areas like islands. The use of chemicals (*i*) has adverse effects on the environment and health and (*ii*) can led to the development of resistance in mosquito populations [58,59]. In this context, only an integrated management combining these methods can carry out to an efficient and durable vector control strategy [21]. However, parameters of these optimal strategies (time of application, duration, number and frequency of the treatments, *etc.*) are complex to determine. Thus, modeling approaches can be helpful to test and compare mosquito population reduction methods, before long and expensive operational field trials. These studies should address the compared efficiency of control tools according to different conditions of use. Special attention should be paid to the optimal time of application: indeed, if insecticides treatments are usually started and repeated during the second semester of the year, when mosquito abundance is high in the field [21], modeling studies suggest that applying treatments earlier—in spring—may be more efficient [17]. Modeling approaches should also address other conditions of use such as the number of traps, treatments frequencies, the minimal percentage of the targeted mosquito population affected by the treatment, or the level of maintenance of community implication required to reduce mosquito population. 

Another perspective of the use of our model concerns the assimilation of the predicted abundance of host-seeking mosquitoes into a predictive model of host-vector contacts taking into account the human population density and exposure [60]. Such an approach will provide maps of the entomological risk induced by *Ae. albopictus*, taking into account the seasonal variations of host and vector distributions. The *Ae. albopictus* dynamics model could also be linked to an epidemiological model of transmission, such as compartmental models [14,61] or agent-based models [62], to study in more details the possible spread of dengue or chikungunya viruses. 

## 4. Conclusions

The population dynamics of *Ae. albopictus* in a temperate climate environment have been modelled for the first time, using a mechanistic approach. The model, driven by temperature and rainfall, correctly predicted entomological field data of egg stage over a four year period. It can be used efficiently as a tool to predict *Ae. albopictus* population dynamics, and to assess the efficiency of different control strategies.
